# Supplementary material for: Beneficial Effects of Pulmonary Vasodilators on Pre-Capillary Pulmonary Hypertension in Patients with Chronic Kidney Disease on Hemodialysis
Source: Life (Basel). 2022 May 24;12(6):780. doi: 10.3390/life12060780 (PMC9224627; doi:10.3390/life12060780)
Supplement: Supplementary file 1 [file life-12-00780-s001.zip › life-1686454-supplementary.pdf]

Table S1. Demographic and clinical data of the studied patients (n = 7).

Patient No.		1	2	3	4	5	6	7
Outcome measures and catheterization parameters								
Dry weight (kg)	Before	52.8	52.3	48.0	76.5	55.6	40.5	45.0
	On drugs	52.0	49.6	46	81.0	54.1	50.1	45.5
BNP (pg/mL)	Before	No data	1217	655	783	1201	603	623
	On drugs	7	238	447	44	140	595	1080
WHO functional class	Before	III	III	III	III	III	III	III
	On drugs	II	II	II	I	II	II	II
6-minute walk distance (meter)	Before	180	No data	360	No data	186	No data	380
	On drugs	550	No data	490	390	590	No data	470
Mean artery pressure (mmHg)	Before	50	101	56	100	90	88	102
	On drugs	93	90	65	61	71	93	115
Heart rate (bpm)	Before	89	60	75	105	90	76	88
	On drugs	82	69	61	90	65	69	71
Mean pulmonary artery pressure (mmHg)	Before	37	30	34	35	32	36	50
	On drugs	16	32	27	22	33	39	54
Diastolic pulmonary artery pressure (mmHg)	Before	24	17	20	25	24	23	33
	On drugs	12	20	14	11	19	25	32
Mean pulmonary capillary wedge pressure (mmHg)	Before	5	8	10	10	4	14	11
	On drugs	7	17	15	7	9	27	11
Diastolic pressure gradient (mmHg)	Before	19	9	10	15	20	9	22
	On drugs	5	3	-1	4	10	-2	21
Pulmonary vascular resistance (wood units)	Before	7.8	5.6	5.4	5.4	11.2	7.4	5.9
	On drugs	2.1	3.2	2.5	3.3	3.1	2.6	8.5
Cardiac Index (L/min/m^2)	Before	2.2	2.8	3.2	2.9	1.6	3.0	4.1
	On drugs	3.7	3.2	3.5	2.9	4.6	3.1	3.2
